# Comparing Causal Bayesian Networks Estimated from Data

**DOI:** 10.3390/e26030228

**Published:** 2024-03-02

**Authors:** Sisi Ma, Roshan Tourani

**Affiliations:** Institute for Health Informatics, University of Minnesota, Minneapolis, MN 55455, USA

**Keywords:** causal Bayesian network, causal discovery, uncertainty, resampling

## Abstract

The knowledge of the causal mechanisms underlying one single system may not be sufficient to answer certain questions. One can gain additional insights from comparing and contrasting the causal mechanisms underlying multiple systems and uncovering consistent and distinct causal relationships. For example, discovering common molecular mechanisms among different diseases can lead to drug repurposing. The problem of comparing causal mechanisms among multiple systems is non-trivial, since the causal mechanisms are usually unknown and need to be estimated from data. If we estimate the causal mechanisms from data generated from different systems and directly compare them (the naive method), the result can be sub-optimal. This is especially true if the data generated by the different systems differ substantially with respect to their sample sizes. In this case, the quality of the estimated causal mechanisms for the different systems will differ, which can in turn affect the accuracy of the estimated similarities and differences among the systems via the naive method. To mitigate this problem, we introduced the bootstrap estimation and the equal sample size resampling estimation method for estimating the difference between causal networks. Both of these methods use resampling to assess the confidence of the estimation. We compared these methods with the naive method in a set of systematically simulated experimental conditions with a variety of network structures and sample sizes, and using different performance metrics. We also evaluated these methods on various real-world biomedical datasets covering a wide range of data designs.

## 1. Introduction

In biomedical sciences, sometimes the researchers are interested not only in the causal mechanisms underlying one system but also in how the causal mechanisms may be consistent or distinct among several systems. This comparative information can improve the understanding of the individual systems in question and can indicate effective interventions. For example, consistent molecular pathways underlying distinct cancers of different organs or between cancer and other diseases can be an indication for repurposing existing effective therapeutics [1,2]. On the other hand, the increasing knowledge of the differences between the neural mechanisms of the healthy population vs. the population with Parkinson’s disease has led to improved treatment strategies using deep brain stimulation [3,4,5]. Moreover, with the rapid developments in measurement technology, the collection of multi-modular, high volume, and/or high-intensity longitudinal data has become more economical in many health domains. Deriving and comparing individualized causal mechanisms could inform precision and personalized medicine [6,7,8].

The discovery and comparison of causal mechanisms can be and is often achieved through conducting randomized experiments and analyzing experimental data. However, experiments are often costly, time-consuming, sometimes unethical, or even outright impossible, especially in the biomedical domain. In contrast, observational data are often more abundant and cost-effective to collect. Various methods, generally referred to as computational causal discovery methods, have been developed for estimating the structure of causal networks based on the statistical properties of observational data. These methods can be entirely data-driven. They use observational data, experimental data, or a mixture of both as inputs. A variety of prior knowledge regarding the domain can also be incorporated. The correctness of these methods has been proven under broad assumptions [9,10]. In the past ten years, there has been an accelerated growth in the application of these methods to biological and medical data for knowledge discovery, which has achieved promising results [11,12,13,14,15].

However, using computational causal discovery methods for the *comparison of causal networks* is more complicated than simply applying the causal discovery method to each dataset, respectively, and comparing the resulting networks (we refer to this procedure as the naive method). This is because, in general, the quality of the causal network discovery depends on various factors, and the naive method does not capture the confidence of the estimation.

The current study aims to answer the following questions: What are some methods for comparing causal networks estimated from different datasets? What are their comparative performances under different conditions? What sample sizes are sufficient to support causal network comparison? What are the factors influencing the performance of causal network comparison? Finally, do these factors interact with one another? We systematically explore these questions with analytical experiments on simulated data and various real-world data. We introduce and examine three network comparison methods and characterize their performance under various sample sizes, network structure characteristics, and effect sizes over a comprehensive collection of performance metrics.

The organization of the paper is as follows. In Section 2, we review the key concepts in computational causal discovery. We then formulate the causal network comparison problem, define the scope of the current study, and review relevant prior literature. In Section 3, we describe three methods for causal network comparison, provide an illustrative example showcasing their application, and introduce metrics for evaluating these methods. In Section 5 and Section 6, we evaluate the network comparison methods on simulated and real-world data, respectively. We present the design of the analytical experiments, analyze the results, and discuss their implications. In Section 7, key findings from the analytical experiements are summarized. Lastly, in Section 8 we discuss the contributions and limitations of the current work. We also point to several directions for future work.

## 2. Background, Problem Formulation, and Scope

We first briefly introduce computational causal discovery. Then, we present the general problem formulation for comparing a pair of causal Bayesian networks. We then describe the scope of the current paper, which is the comparison of causal structures.

### 2.1. Computational Causal Discovery

Computational causal discovery solves the general problem of discovering qualitative and quantitative causal relationships from data. Qualitative causal relationships describe the existence or absence of cause–effect relationships, i.e., the causal structure among variables; e.g., whether the over-expression of gene X causes cancer or not. The problem of discovering qualitative relationships is often referred to as causal structure discovery. On the other hand, quantitative causal relationships describe the magnitude of impact a cause has on its effect, e.g., how much cardiovascular risk will be reduced if the dose of a medication is increased. The problem of discovering quantitative causal relationships is often referred to as causal inference or causal effect estimation. In general, the causal effect estimation will be biased if the causal structure between the relevant variables is not correctly specified [9]. In the current study, we focus on the comparison between causal structures.

We have only introduced the essential concepts of causal structure discovery for brevity in this section. We refer the reader to the following sources for a more in-depth introduction to the topic [9,10,16,17].

#### 2.1.1. Definitions

Herein, we introduce the definitions for causality, a Bayesian network, and a causal Bayesian network. The first two definitions give rise to the latter, which represents a system’s causal mechanisms.

We use the definition for causality associated with manipulation or experimentation. The do(·) notation in Definition 1 refers to a manipulation or experimentation that fixes the values of random variable *X* to a single value *x*.

**Definition** **1.** *Causation [9]. Let $\mathrm{do}(X=x_{i})$ denote a manipulation, where the value of X is set to $x_{i}$. If $\exists x_{i},x_{j}$, such that $p\left(Y|\mathrm{do}\left(X=x_{i}\right)\right)\neq p\left(Y|\mathrm{do}\left(X=x_{j}\right)\right)$, then X is a cause of Y.*

We use a definition for a Bayesian network [18] with modified notation, which is suitable for later discussions of network difference.

**Definition** **2.** *Bayesian network. Let V be a set of variables and p be a joint probability distribution over V. Let E be the set of edges of a directed acyclic graph (DAG), where all vertices of the DAG correspond one-to-one to members of V. ∀X∈V, X is conditionally independent of all non-descendants of X, given the parents of X (i.e., the Markov condition holds). The triplet 〈V,E,p〉 defines a Bayesian network.*

Taking the above two definitions together, a causal Bayesian network is a Bayesian network with causally relevant edge semantics. In a causal Bayesian network, the parents of variable *X* are the direct causes of *X*, the children of *X* are direct effects of *X*, the non-parent ancestors of *X* are indirect causes of *X*, and the non-children descendants of *X* are indirect effects of *X*.

**Definition** **3.** *Causal Bayesian Network [9,10]. A causal Bayesian network 〈V,E,p〉 is the Bayesian network 〈V,E,p〉 with the additional semantics that, if there is an edge X→Y in E, then X directly causes Y, ∀X,Y∈V.*

#### 2.1.2. The Causal Structure Discovery Problem

We formulate the causal structure discovery problem as follows. Let G〈V,E,p〉 denote a causal Bayesian network over a set of variable V with the joint distribution *p*. We introduce *E*, an equivalent representation of the edge set E for notational convenience. *E* represents the relationship between a pair of variables *X* and *Y*∈V with E:{(X,Y)|X,Y∈V}→{0,1}, where value 1 indicates the presence of a direct causal link X→Y, and value 0 indicates the absence of such a relationship. In other words, *E* is the adjacency matrix representing the structure of *G*. Let *D* be a dataset generated from *G* with the sample size *N*.

The causal discovery problem is to estimate *G* from *D*. Let $\hat{G}$ be the causal Bayesian network inferred from *D*; the estimated network is similarly defined as $\hat{G}\langle V,\hat{E},\hat{p}\rangle$, except for $\hat{E}:\left\{\left(X,Y\right)|X,Y\in V\right\}\to \left[0,1\right]$. We allow the value assigned to X→Y to range between zero and one, to account for uncertainty or variability in the estimation when applicable. We expand on this point in Section 3.

#### 2.1.3. Methods for Causal Structure Discovery

In general, causal relationships can not be discovered from observational data without assumptions [9,19]. Many methods, (including arguably the three most studied and applied methods, the PC (Peter-Clark), FCI (Fast Causal Inference) [10], and GES (Greedy Equivalence Search) [20]) assume faithfulness. The faithfulness assumption establishes a one-to-one correspondence between the structure of the data generation process (i.e., the causal structure) and the statistical properties of the data, thus making causal discovery from observational data possible. Several more recent methods have sought to discover causal structure under weaker versions of faithfulness or specific types of faithfulness violation [21,22,23,24].

Causal structure discovery methods for causal structure discovery from data are generally categorized into two broad categories: the constraint-based methods and the score-based methods. The constraint-based methods search for the causal structure underlying the data, based on statistical constraints imposed by conditional independence relationships estimated from data. Examples of constraint-based methods include the PC algorithm and the FCI algorithm [10]. The score-based methods instead search for the causal structure underlying the data by maximizing likelihood-based scores. An example of a score-based algorithm is the GES algorithm [20,25]. Additionally, there are hybrid methods that utilize ideas and techniques from both constraint-based and score-based methods, such as the MMHC (Max-Min Hill-Climbing) [26] and GFCI [27].

### 2.2. The Causal Network Comparison Problem

The causal network comparison problem deals with the general issue of comparing causal Bayesian networks, given data generated from them. This problem is the focus of the current study. Below, we discuss the general mathematical formulation of this problem, the specific aspects of the problem that the current study addresses, and relevant prior literature.

#### 2.2.1. General Definition

We formulate the general problem of causal network comparison as inferring the difference between an ordered pair of true networks $\left(G_{i},G_{j}\right)$ given the inferred network $\left(\hat{G_{i}},\hat{G_{j}}\right)$ derived from the pair of datasets $\left(D_{i},D_{j}\right)$ using method M.

#### 2.2.2. Differences between Networks

A pair of networks $G_{i},G_{j}$ can be compared in many ways. Different metrics are needed to quantify the similarities and differences between networks, depending on the study goal. Performance measures for network comparison can be categorized as performance measures for causal structure comparison and for causal effect comparison. Metrics for causal structure comparison quantify the differences between two causal structures (qualitative causal relationships, such as if the edge X→Y is in $G_{i}$ and $G_{j}$), without taking into account the specific functional form or parameterization of the causal relationships embedded in the joint distribution *p*. Contrastingly, the performance measurements for causal effect comparison capture the quantitative difference in causal effect; namely, if the estimated effect of manipulating *X* on *Y* differs in $G_{i}$ vs. $G_{j}$. The estimated effect is not only related to the structure but also the functional form or parameterization of the networks.

In the current study, we investigate causal structure comparison exclusively; specifically, we define the difference in causal structure between the pair of network $\left(G_{i},G_{j}\right)$ as the set of edges in $G_{i}$ but not in $G_{j}$. For notational convenience and the ease of describing the metrics for quantifying causal structure comparison, we represent the edge difference as follows:

(1) $$\left[E_{i}-E_{j}\right]\left(\left(X,Y\right)\right)=\left\{\begin{array}{cc} 1 & \mathrm{if}\;E_{i}\left(\left(X,Y\right)\right)-E_{j}\left(\left(X,Y\right)\right)=1\\ 0 & \mathrm{Otherwise} \end{array}\right.$$

∀(X,Y)∈V.

X→Y is in $G_{i}$ but not $G_{j}$. $E_{i}\left(\left(X,Y\right)\right)-E_{j}\left(\left(X,Y\right)\right)=0$ indicates X→Y is either in both $G_{i}$ and $G_{j}$, in neither $G_{i}$ nor $G_{j}$, or not in $G_{i}$ but in $G_{j}$. This definition enables us to view the problem of estimating structural differences between pairs of networks as a binary classification problem and to use the performance measurements for binary classification to estimate the performance for this task; further discussion can be found in Section 4.

In addition to evaluating network differences as defined in (1), i.e., the differences in the estimated directed acyclic graph (referred to as orientation discovery performance below), we also evaluated the difference in the presence and absence of the edges and disregarded the directionality of the edge (i.e., comparisons were made based on the skeleton of the causal Bayesian network, referred to as skeleton discovery performance below):

(2) $$\left[E_{i}-E_{j}\right]\left(\langle X,Y\rangle \right)=\left\{\begin{array}{cc} 1 & \mathrm{if}\;E_{i}\left(\langle X,Y\rangle \right)-E_{j}\left(\langle X,Y\rangle \right)=1\\ 0 & \mathrm{Otherwise} \end{array}\right.$$

In other words, $E_{i}\left(\langle X,Y\rangle \right)-E_{j}\left(\langle X,Y\rangle \right)=1$ indicates that an edge, regardless of directionality, exists between *X* and *Y* in $G_{i}$ but not in $G_{j}$. Note that the difference between Equations (1) and (2) is that the former is defined for an ordered pair (X,Y), but the latter is defined for an unordered pair 〈X,Y〉; in other words, $E_{i}\left(\langle X,Y\rangle \right)=E_{i}\left(\langle Y,X\rangle \right)$.

#### 2.2.3. Relevant Prior Literature

The causal network comparison problem as defined here (i.e., the comparison of two networks inferred from data) bears similarity to the problem of evaluating the quality of causal discovery, where the inferred network is compared to the true network. The difference between the inferred network and the true network is regularly evaluated in studies aiming to assess the performance of causal structure discovery methods given simulated data, or when the data generation function is known [28,29]. Despite the similarity on the surface, comparing two networks inferred from data is more challenging, since both networks in question were estimated and may have different degrees of uncertainty associated with them.

The comparison of two inferred causal networks belongs to the more general problem of comparing a pair of inferred statistics. On a high level, this comparison is done by assessing the overlap between the confidence intervals of the estimates. Closed-form formulas or approximations for confidence intervals exist for some estimates of interest, such as the mean [30], variance [31], and correlation coefficients [32,33]. However, to the best of our knowledge, a closed-form formula for the confidence interval for causal structure discovery has not been established.

Estimating confidence interval for causal discovery is related to prior work on controlling the false discovery rate for causal discovery, since the control of the false discovery rate requires assigning uncertainty or confidence to the discovery edges. Several methods tackling this problem combine and bound *p*-values from conditional independence tests associated with a particular discovered edge, and then apply a false discovery rate control method over the bounded *p*-values for all discovered edges [34,35,36]. These methods, due to the need to combine *p*-values for bounds, are specific for the PC algorithm variant in question and may not be straight-forward to generalize to other methods. Other methods for estimating the false discovery rate circumvent the bounding *p*-values by employing permutation [35,37] or resampling [35]. In principle, permutation and resampling methods do not depend on the discovery methods applied and/or the knowledge of the joint distribution. The resampling methods are directly related to the goal of the current study, except we are not only interested in bootstrap frequency for the discovered edges (which relates to false discovery rate), but also the pair of nodes for which no edge was identified between them (relates to false negatives). The resampling was first proposed for assessing confidence intervals for causal discovery in [38,39], and was empirically demonstrated to adequate estimate confidence for causal discovery in simulated datasets using various causal discovery algorithms [38,39,40,41,42]. Therefore, we resort to resampling to estimate the confidence of causal discovery and to support the comparison between the inferred networks.

Other relevant works come from the applied literature. Many studies in the biomedical literature compare networks derived from data collected from different populations. Take studies using fMRI (functional magnetic resonance imaging) data, for example; many studies compare the networks derived from fMRI data, collected from individuals affected by a given disease, with controls [43,44,45,46]. Except for [44], all of these studies derive connectivity networks. The connectivity networks capture associations rather than causation and compare connectivity network results in conclusions regarding differences in univariate statistical association rather than mechanistic differences. In addition, all studies are either based on fMRI data of the same sample size for each individual or do not mention potential sample size differences. For fMRI data, especially for resting state data (examined in all four studies), it is relatively easy to enforce equal sample size for network discovery. However, this is not generally true for data from other domains of medicine. In the current study, the differential confidence of estimation due to sample size difference is one of the critical challenges we tackled.

## 3. Methods for Estimating the Structural Differences between Pairs of Networks

In this section, we design and introduce three methods for estimating the structural differences between pairs of causal Bayesian networks and illustrate their differences with a simple example.

### 3.1. Naive Method

Given a pair of datasets $\left(D_{i},D_{j}\right)$ generated by $\left(G_{i},G_{j}\right)$, obtain $\left({\hat{G_{i}}}^{naive},{\hat{G_{j}}}^{naive}\right)$ by applying the causal discovery algorithm of choice M to $D_{i}$ and $D_{j}$, respectively. The oriented edge difference between $G_{i}$ and $G_{j}$, i.e. $E_{i}-E_{j}$, is estimated by ${\hat{E_{i}}}^{naive}-{\hat{E_{j}}}^{naive}:\left(\left(X,Y\right)\right)\to \left\{0,1\right\}$, where:

(3) $$\left[{\hat{E_{i}}}^{naive}-{\hat{E_{j}}}^{naive}\right]\left(\left(X,Y\right)\right)=\left\{\begin{array}{cc} 1 & \mathrm{if}\;{\hat{E_{i}}}^{naive}\left(\left(X,Y\right)\right)-{\hat{E_{j}}}^{naive}\left(\left(X,Y\right)\right)=1\\ 0 & \mathrm{Otherwise} \end{array}\right.$$

In other words, ${\hat{E_{i}}}^{naive}\left(\left(X,Y\right)\right)-{\hat{E_{j}}}^{naive}\left(\left(X,Y\right)\right)=1$ indicates that the naive method estimates the existence of the edge X→Y, in $G_{i}$ but not in $G_{j}$. The estimated skeleton or unoriented edge difference $\left[{\hat{E_{i}}}^{naive}-{\hat{E_{j}}}^{naive}\right]\left(\langle X,Y\rangle \right)$ is defined similarly and is, thus, omitted.

The naive method is simple and easy to implement. However, as is the same for any statistical procedure, sample sizes of $D_{i}$ and $D_{j}$ impact the estimation of $G_{i}$ and $G_{j}$. More importantly for our problem, if there is a sufficient difference in the sample sizes of $D_{i}$ and $D_{j}$, the quality for estimating $E_{i}$ and $E_{j}$ will be different, i.e., how well $\hat{E_{i}}$ approximates $E_{i}$ vs. how well $\hat{E_{j}}$ approximates $E_{j}$, which will further impact how well $\hat{E_{i}}-\hat{E_{j}}$ approximates $E_{i}-E_{j}$. In the following sections, we introduce the bootstrap estimation and the equal sample size resampling estimation, with the goal of mitigating this problem.

### 3.2. Bootstrap Estimation

Bootstrap is often used to assess the variability in an estimation. In the causal discovery literature, the frequency of discovering an edge in bootstrap samples has been shown to be a good indicator for the presence of the edge in the true network [42]. Therefore, we propose to estimate the network difference by incorporating the confidence of estimating individual networks using bootstrap. Specifically, we apply the causal discovery algorithm of choice to bootstrap samples of $D_{i}$ and $D_{j}$ respectively, and obtain for each edge the bootstrap percentage (the number of times an edge is discovered in a bootstrap sample over the total number of bootstrap runs) ${\hat{E_{i}}}^{BS}$ and ${\hat{E_{j}}}^{BS}$. Here, ${\hat{E}}^{BS}$ returns a value in [0,1]. The edge difference for $\left(G_{i},G_{j}\right)$ is estimated by ${\hat{E_{i}}}^{BS}-{\hat{E_{j}}}^{BS}:\left(X,Y\right)\to \left[-1,1\right]$, where

(4) $$\left[{\hat{E_{i}}}^{BS}-{\hat{E_{j}}}^{BS}\right]\left(\left(X,Y\right)\right)={\hat{E_{i}}}^{BS}\left(\left(X,Y\right)\right)-{\hat{E_{j}}}^{BS}\left(\left(X,Y\right)\right),\;\forall X,Y\in V$$

Heuristically, the larger the ${E_{i}}^{BS}-{\hat{E_{j}}}^{BS}$ for a given edge, the more likely it is to be present in $G_{i}$ but not in $G_{j}$.

### 3.3. Equal Sample Size Resampling Estimation

Equal sample size resampling estimation is, in principle, similar to the bootstrap estimation. The difference is that, instead of obtaining the bootstrap probability estimation from both $D_{i}$ and $D_{j}$, we obtain the bootstrap probability estimation from the dataset with the smaller sample size and obtain the equal sample size resampling estimation from the dataset with the larger sample size. The equal sample size resampling down-samples the dataset with the larger sample size without replacement, to create subsamples of the same size as the dataset with the smaller sample size. The causal discovery algorithm of choice is applied to the equal sample size resampling samples and bootstrap samples of the two datasets, respectively. The edge difference for $\left(G_{i},G_{j}\right)$ is estimated by ${\hat{E_{i}}}^{RSBS}-{\hat{E_{j}}}^{RSBS}:\left(X,Y\right)\to \left[-1,1\right]$, where

(5) $$\left[{\hat{E_{i}}}^{RSBS}-{\hat{E_{j}}}^{RSBS}\right]\left(\left(X,Y\right)\right)=\left\{\begin{array}{cc} {\hat{E_{i}}}^{RS}\left(\left(X,Y\right)\right)-{\hat{E_{j}}}^{BS}\left(\left(X,Y\right)\right) & \mathrm{if}\;\mathrm{sample}\;\mathrm{size}\;\mathrm{of}\;D_{i}\;\mathrm{is}\;\mathrm{larger}\\ {\hat{E_{i}}}^{BS}\left(\left(X,Y\right)\right)-{\hat{E_{j}}}^{RS}\left(\left(X,Y\right)\right) & \mathrm{if}\;\mathrm{sample}\;\mathrm{size}\;\mathrm{of}\;D_{j}\;\mathrm{is}\;\mathrm{larger} \end{array}\right.$$

Heuristically, the larger the ${\hat{E_{i}}}^{RSBS}-{\hat{E_{j}}}^{RSBS}$ for a given edge, the more likely it is to be present in $G_{i}$ but not in $G_{j}$.

The advantage of equal sample size resampling over bootstrap resampling are with respect to edges that are present in both $G_{i}$ and $G_{j}$, but there is enough statistical power to identify the edge for one but not the other due to the sample size difference.

### 3.4. Example

In this section, we show a simple example to illustrate the three methods for estimating network differences and highlight their advantages and disadvantages. We illustrate the network difference in the skeleton difference, but it can be easily extended to orientation difference.

**Figure 1:**
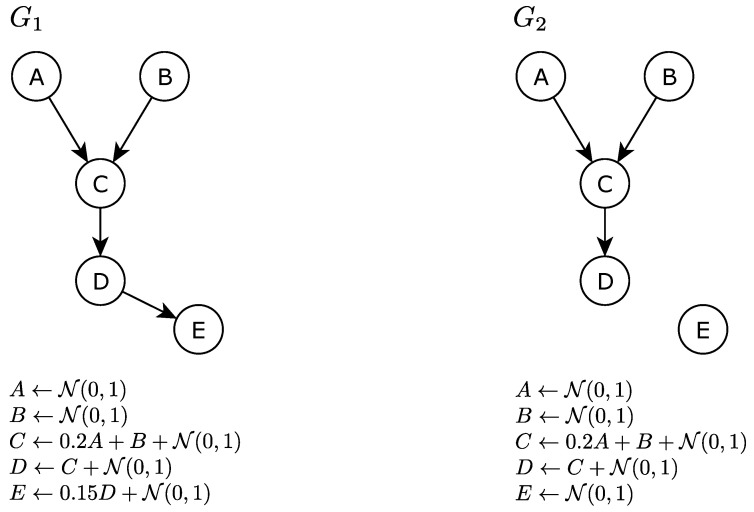
A pair of networks $\langle G_{i},G_{j}\rangle$ and their associated data generation functions.

The true causal structure for a pair of networks $\left(G_{1},G_{2}\right)$ is shown in Figure 1. The two networks and their associated data generation functions are identical, except for the edge between *D* and *E*. The true edge difference $E_{1}-E_{2}$ only contains one edge, which is *D*—*E*. We generated $D_{1}$ with 1000 samples from $G_{1}$, and $D_{2}$ with 200 samples from $G_{2}$. We applied the PC algorithm with the three methods for estimating network difference $E_{1}-E_{2}$, and obtained the results, as can be seen in Table 1.

The naive method estimated $G_{1}$ perfectly, but missed the *A*—*C* edge for $G_{2}$. As a result, it assigned a value of one to *D*—*E* and *A*—*C* for the estimated network difference ${\hat{E_{1}}}^{naive}-{\hat{E_{2}}}^{naive}$.

The bootstrap method identified *A*—*C* 86 and 10 percent of the time out of all the bootstrap runs, when estimating $E_{1}$ and $E_{2}$, respectively. As a result, it assigned the value 0.76 to the *A*—*C* edge for ${\hat{E_{1}}}^{BS}-{\hat{E_{2}}}^{BS}$. Notice that, due to bootstrap’s ability to assess variability over multiple bootstrap samples, the ${\hat{E_{1}}}^{BS}-{\hat{E_{2}}}^{BS}$ for the true different edge *D*—*E* is higher than that of *A*—*C*, which is desirable. However, the bootstrap method also assigned relatively small but positive estimates to two edges, *A*—*D* and *B*—*E*, which are not in $E_{1}-E_{2}$.

The equal sample size resampling method subsampled $D_{1}$ with the same sample size as $D_{2}$ to obtain ${\hat{E_{1}}}^{RS}$, resulting in less confidence, as represented by a value of 0.16 for the *A*—*C* edge. The ${\hat{E_{1}}}^{RSBS}-{\hat{E_{2}}}^{RSBS}$ for *A*—*C* estimated by the equal sample size resampling is 0.06, a much smaller number compared to the other two methods, and the estimated value is fairly close to other edges that are not in $E_{1}-E_{2}$, which is desirable. However, the ${\hat{E_{1}}}^{RSBS}-{\hat{E_{2}}}^{RSBS}$ for the true different edge *D*—*E* is 0.42, a value smaller than those generated by the naive method and the bootstrap method.

To summarize, both the bootstrap and equal sample size resampling methods incorporate estimations of variability, which allowed for distinction between the true different edge *D*—*E* and the edges that are not different for $\left(G_{1},G_{2}\right)$, most notably *A*—*C*. As a result, in this example, there exist thresholds (e.g., >0.76 for bootstrap and >0.06 for equal sample size resampling) where both the bootstrap method and the equal sample size resampling method can result in perfect discovery performance for $E_{1}-E_{2}$. Comparing bootstrap with equal sample size resampling, the bootstrap tends to result in larger $\hat{E_{1}}-\hat{E_{2}}$ for all edges in this example, where the sample size of $D_{1}$ is much larger than $D_{2}$. Depending on the characteristics of $G_{1}$ and $G_{2}$ (e.g., structure, effect size of edges, and how many edges are different) and the sample size, the bootstrap and equal sample size resampling methods can have different comparative advantages. We systematically explore this in Section 5.

## 4. Performance Measures

As defined in Section 2, the ground truth for the $\left(G_{i},G_{j}\right)$ edge difference is defined by $E_{i}-E_{j}:\left(X,Y\right)\to \left\{0,1\right\}$. This formulation enables us to treat the estimation of edge difference as a binary classification problem. Positives are edges where $E_{i}-E_{j}$ takes a value of one, i.e., edges that are in $G_{i}$ but not $G_{j}$. Negatives are edges where $E_{i}-E_{j}$ takes a value of zero, i.e., edges that are in both $G_{i}$ and $G_{j}$, edges that are in neither $G_{i}$ nor $G_{j}$, or edges that are in $G_{j}$ but not $G_{i}$. Note that the edge differences for $\left(G_{i},G_{j}\right)$ and $\left(G_{j},G_{i}\right)$ are distinct.

We use standard metrics for binary classification to evaluate the performance for estimating network difference. The naive method (Equation (3)) outputs a binary decision. We compute AUCROC (area under the receiver operating characteristic curve), AUPR (area under the precision recall curve), and cross entropy for all three estimation methods. For the naive methods, we directly compute metrics for evaluation, including sensitivity, specificity, PPV (positive predictive value), NPV (negative predictive value), $F_{1}$ score, and accuracy. For the bootstrap estimation (Equation (4)) and equal sample size resampling estimation (Equation (5)) we thresholded/binarized the heuristic score so the binary classification metrics can be computed. The threshold is obtained by optimizing the $F_{1}$ score. We will focus on comparing AUCROC, AUPR, and cross entropy in the main body of the paper, but we report the other metrics in the Supporting Information.

In binary classification, it is often reasonable to let each observation contribute to the performance equally, but, in a network, edges can play different roles, and the discovery of edges can depend on other edges. Here, to keep the our discussion clear, we used the standard performance measurements for binary classification where each observation is weighted equally. But, depending on the goal of the study, it might be beneficial to treat individual edges differently, e.g., to focus on a subgraph of interest.

In general, the stronger the direct causal relationship among two variables in the true network, the easier it is to identify the relationship. Recall the example shown in Figure 1 and Table 1: edge *A*—*C* is difficult to discover at a smaller sample size when compared to *B*—*C*, due to the weaker edge strength. The strength of the direct causal relationship is often referred to as the effect size. To examine the influence of effect size on the performance for identifying network differences, we correlated edge effect sizes with the heuristic scores from the bootstrap and equal sample size resampling. The effect size for edge X→Y is defined as $f_{XY}^{2}=\frac{{R^{2}}_{\mathrm{Pa}(Y)}-{R^{2}}_{\left(\mathrm{Pa}(Y)\setminus X\right)}}{1-{R^{2}}_{\mathrm{Pa}(Y)}}$, where Pa(Y) denotes the set that contains all parents of *Y* in $G_{i}$. $f_{XY}^{2}$ is interpreted as the additional information in *X* regarding *Y*, given other parents of *Y* [47].

## 5. Experiments with Simulated Data

To systematically investigate the factors influencing the quality of network difference inference, we generated pairs of causal Bayesian networks $\left(G_{i},G_{j}\right)$ with different edge densities, different edge strengths (effect sizes), and different numbers of edges differences between the pair. We also simulated datasets of different sizes from the simulated networks, to assess the effect of the sample size. We then applied the three edge difference discovery methods to the simulated datasets, to investigate how the above factors influence the performance.

### 5.1. Simulation Procedure

Let $N_{v}$ denote the number of variables, $N_{e}$ denote the number of edges, and $N_{d}$ denote the number of different edges between the pair of networks $\left(G_{i},G_{j}\right)$. We generate the graphs, such that, $N_{d}=2|\left\{\left(X,Y\right)|E_{i}-E_{j}=1\right\}\left|\;=\;2\right|\left\{\left(X,Y\right)|E_{j}-E_{i}=1\right\}|$, where $\left\{\left(X,Y\right)|E_{i}-E_{j}=1\right\}$ denote the set of edges that are in $G_{i}$ but not in $G_{j}$. In other words, we simulate the number of edges that are in $G_{i}$ but not $G_{j}$ to be equal to the number of edges that are in $G_{j}$ but not $G_{i}$; that is, $\frac{N_{d}}{2}$ for each.

To generate a pair of causal Bayesian networks $\left(G_{1},G_{2}\right)$, we first generated $G_{1}$ by generating a random directed acyclic graph (DAG) with $N_{v}$ nodes and $N_{e}$ edges. Then, the DAG is parameterized as a multivariate standard Gaussian distribution, as follows:

(6) $$V_{i}=\left\{\begin{array}{cc} N(0,1) & \mathrm{if}\;\mathrm{Pa}(V_{i})=\emptyset \\ \Sigma_{V_{p}\in \mathrm{Pa}\left(V_{i}\right)}\beta_{p}V_{p}+N\left(0,\sigma_{noise}\right) & \mathrm{if}\;\mathrm{Pa}(V_{i})\neq \emptyset \end{array}\right.$$

$\mathrm{Pa}(V_{i})$ represents the set containing the parents of $V_{i}$, as specified in the DAG. $\beta_{p}$, the coefficient of each parent of $V_{i}$, is the multiplication of a uniform random variable and a Bernoulli random variable, as follows: $\beta_{p}=b\times u$, where P(b=1)=0.6, P(b=−1)=0.4, and u∼U(0.1,0.35). This procedure resulted both positive and negative relationships in the data generation process and a range of effect sizes (for the distribution of the effect sizes, see the Supporting Information). The effect sizes explored in the current study are mainly small ($f^{2}\in \left[0.02,0.15\right)$) to median ($f^{2}\in \left[0.15,0.35\right)$) effect sizes [47,48]. $\sigma_{noise}$ is computed for each $V_{i}$, such that the marginal variance of $V_{i}$ is one. A marginal variance of one is not always achievable (i.e., in some cases, the variance of $V_{i}$ exceeds one before $\sigma_{noise}$ is added); in these cases, a new DAG is generated and new parameterization is attempted.

We generate $G_{2}$ by randomly deleting $\frac{N_{d}}{2}$ edges from $G_{1}$, and randomly adding $\frac{N_{d}}{2}$ edges to $G_{1}$, resulting in $N_{d}$ different edges between $G_{1}$ and $G_{2}$. The edge coefficients that are common between $G_{1}$ and $G_{2}$ have the same coefficients. The edges that were present in $G_{2}$ but not in $G_{1}$ are generated using $\beta_{p}=b\times u$, where P(b=1)=0.6, P(b=−1)=0.4, and u∼U(0.1,0.35). The corresponding $\sigma_{noise}$ terms are recomputed as well, to ensure the marginal distribution of all variables are standard Gaussian for $G_{2}$.

After the parameterized causal Bayesian networks are generated, we simulated datasets of different sample sizes from them.

We explored the following parameters for the simulated causal Bayesian networks: (1) number of nodes: $N_{v}=100$, (2) number of edges: $N_{e}=\left\{1.5\times N_{v},2\times N_{v},2.5\times N_{v}\right\}$, and (3) number of different edges between pairs of networks $N_{d}=\left\{\left\lceil 0.05\times N_{e}\right\rceil ,\left\lceil 0.1\times N_{e}\right\rceil ,\left\lceil 0.2\times N_{e}\right\rceil ,\left\lceil 0.5\times N_{e}\right\rceil ,1\times N_{e}\right\}$. For each parameter combination, we generated 50 random pairs of DAGs and parameterized causal Bayesian networks. We simulated pairs of datasets from each pair of causal Bayesian networks, where the number of samples of datasets simulated from $G_{1}$ are $N_{1}=\left\{500,1000,2000,5000\right\}$. For each sample size of $N_{1}$, we compared the estimated network to datasets simulated from $G_{2}$, with sample sizes of $N_{2}=\left\{0.1\times N_{1},0.2\times N_{1},0.5\times N_{1},1\times N_{1}\right\}$. This resulted in 1×3×5×50×4×4=12,000 pairs of datasets, where we estimated the difference between $\left(G_{1},G_{2}\right)$. For each pair of $\left(G_{i},G_{j}\right)$, we estimated both $E_{1}-E_{2}$ and $E_{2}-E_{1}$. Note that for our setting, data sampled from $G_{1}$ was always larger or equal to that from $G_{2}$.

### 5.2. Performance of Different Methods for Estimating Network Difference

We determined the best method for estimating the difference between two networks by comparing the performances of the three methods for each evaluated outcome, the causal discovery algorithm applied, and different performance measures under each simulation condition. There are a total number of 240 simulation conditions, given the combinations of the number of edges, the number of different edges between the networks, and the number of samples for each network ($|N_{v}\left|\;\times \;\right|N_{e}\left|\;\times \;\right|N_{d}\left|\;\times \;\right|N_{1}\left|\;\times \;\right|N_{2}|\;=240$). There are 50 dataset pairs for each simulation condition for estimations of variance in performance.

Table 2 summarizes the percent of times a network difference estimation method was deemed the best over all the applicable simulation conditions. It is worth noting that the bootstrap method resulted in the best performance over almost all simulation conditions (>90%) for almost all evaluated outcomes, algorithms, and performance measures. The only exception was when assessing the additional oriented edge in the network estimated from a smaller dataset, compared to the network estimated from a larger dataset, using the PC algorithm for the AUCROC (underlined in Table 2). In this situation, the equal sample size resampling method was the best over almost all simulation conditions.

In the following sections, we explore the influence of different factors and their interactions on estimating network differences further.

### 5.3. Effect of Sample Size on Inferring Network Difference

**Figure 2:**
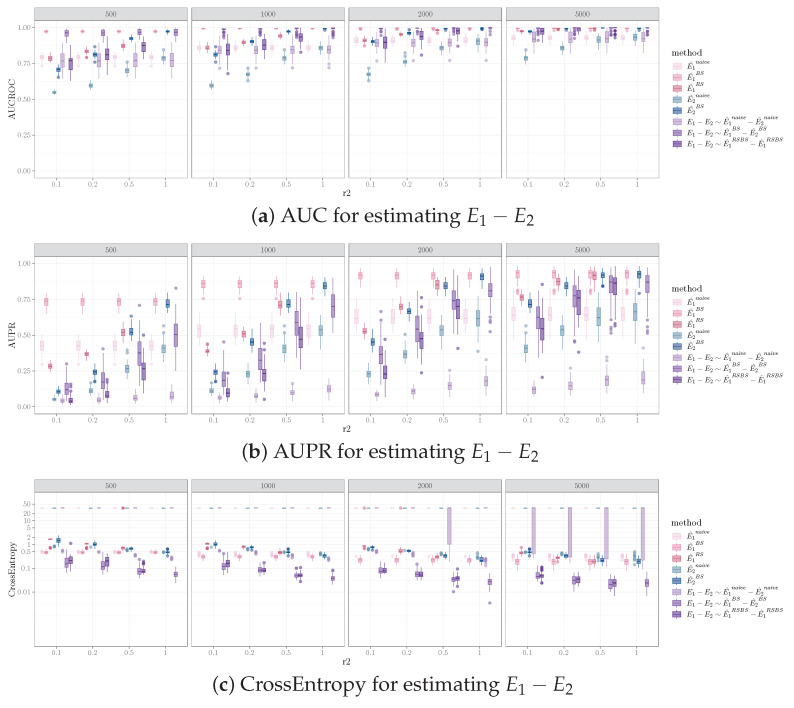
Performance measurements for identifying orientation difference for $E_{1}-E_{2}$ using FGES (Fast Greedy Equivalence Search), where $N_{v}=100$, $N_{e}=200$, $N_{d}=40$. Columns are sample sizes for $D_{1}$, x-axis represents ratio of sample size for $D_{2}$ vs. $D_{1}$. We denote the performance for inferring network differences $E_{1}-E_{2}$ using the naive, bootstrap, and equal sample size resampling methods with different shades of purple. We also denote the performances of estimating $E_{1}$ using the naive, bootstrap, and equal sample size resampling with different shades of pink, and estimating $E_{2}$ using the naive and bootstrap methods with different shades of green. Note that the resampling method is not applicable when the sample sizes of $D_{1}$ and $D_{2}$ are the same.

Increasing the sample size in one sample while holding the sample size for the other sample resulted in performance improvement for identifying different edges between the pairs of networks. The trend of increased performance with increasing sample size was observed for AUCROC, AUPR, and cross entropy for all combinations of the number of edges, the number of different edges in the data generating graphs, and the causal discovery algorithms applied. Figure 2 illustrates this for one simulation set-up, where the pair of graphs $\langle G_{1},G_{2}\rangle$ both have 100 vertices, 200 edges, and 40 different edges (i.e., $N_{v}=100$, $N_{e}=200$, $N_{d}=40$). For a fixed sample size of $D_{1}$ (i.e., data sampled from $G_{1}$, corresponds to one subplot in Figure 2, sample size labeled on top of the gray bar), all performance measurements improved as the sample size for $D_{2}$ (i.e., data sampled from $G_{2}$, corresponds to the x-axis of each subplot in Figure 2, tick label representing $\frac{N_{2}}{N_{1}}$) increased. The influence of sample size on the performance for the bootstrap and equal sample size resampling methods was more pronounced than for the naive method.

It is worth noting that the sample size of the smaller sample (i.e., $D_{2}$) has more impact on the performance, whereas the total sample size of the two samples is less critical. For example, in Figure 2, $N_{1}=500$ and $N_{2}=250$ have better performances than $N_{1}=1000$ and $N_{2}=100$ for AUPR mean (standard deviation) (0.37(0.11) vs. 0.20(0.08) and 0.25(0.08) vs. 0.04(0.17) for bootstrap and equal sample size resampling, respectively) and for cross entropy (0.08(0.04) vs. 0.15(0.09) and 0.08(0.03) vs. 0.17(0.08) for bootstrap and equal sample size resampling, respectively). Also, under certain conditions, when the sample size of the smaller sample is constant, increasing the sample size of the large sample has a relatively small impact on performance. For example, in Figure 2b, comparing $N_{2}=100$ and $N_{1}=500$ vs. $N_{1}=1000$, doubling the sample size of D1 results in marginal to no improvement on AUPR (0.18(0.08) vs.0.20(0.08) and 0.09(0.04) vs. 0.10(0.04) for bootstrap and equal sample size resampling, respectively) and cross entropy (0.15(0.09) vs. 0.15(0.09) and 0.21(0.10) vs. 0.17(0.08) for bootstrap and equal sample size resampling, respectively).

**Figure 3:**
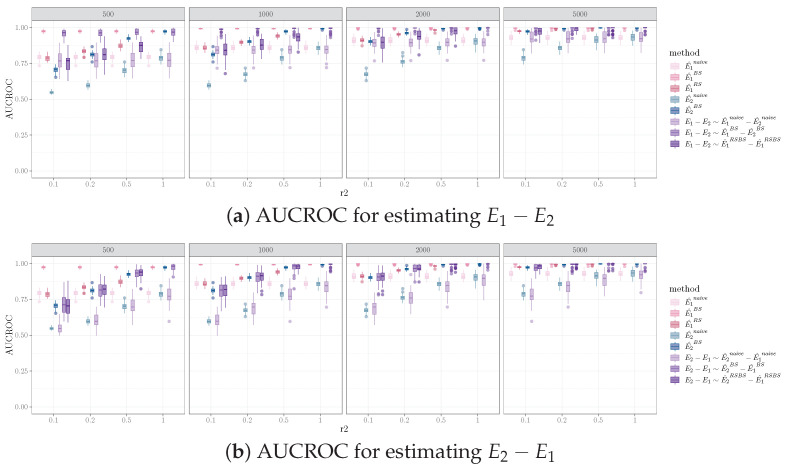
AUCROC for identifying orientation difference for $E_{1}-E_{2}$ vs. $E_{2}-E_{1}$ using FGES, where $N_{v}=100$, $N_{e}=200$, $N_{d}=40$. Columns are sample sizes for $D_{1}$, and the x-axis represents the ratio of sample size for $D_{2}$ vs. $D_{1}$.

Furthermore, given the difference in sample sizes for the two data samples, the performance for identifying $E_{1}-E_{2}$ is different compared to that for $E_{2}-E_{1}$. Specifically, comparing Figure 3a,b, for a fixed sample size ratio $r_{2}=\frac{|D_{2}|}{|D_{1}|}<1$, the AUCROC for $E_{1}-E_{2}$ is higher than that of $E_{2}-E_{1}$, for the naive and bootstrap methods. The advantage for $E_{1}-E_{2}$ diminishes as $r_{2}=\frac{N_{2}}{N_{1}}$ increases. For the equal sample size resampling method, the AUCROC for $E_{1}-E_{2}$ is generally lower than that of $E_{2}-E_{1}$, except for the smaller $D_{1}$ sample size with lower $r_{2}$. This is likely due to the fact that the equal sample size resampling method on $D_{1}$ reduces the identification of true positive edges in $E_{1}$, which decreases the AUCROC for $E_{1}-E_{2}$.

### 5.4. Effect of Causal Discovery Algorithms on Inferring Network Difference

We observed that the PC algorithms resulted in a better performance, compared to FGES, for inferring network differences for most of the simulation set-ups and the performance measures we examined. Among the 2640 combinations of simulation settings (240 for naive and bootstrap, 180 for equal sample size resampling), outcomes evaluated (skeleton and orientation for $E_{1}-E_{2}$ and $E_{2}-E1$, four combinations), and estimation methods (naive, bootstrap, and equal sample size resampling) examined, the PC algorithm performed better or equal to FGES in 98%, 84%, and 92% of the combinations for AUC, AUPR, and cross entropy, respectively. FGES performed better or equal to the PC in 51%, 51%, and 59% of the combinations for AUC, AUPR, and cross entropy, respectively.

**Figure 4:**
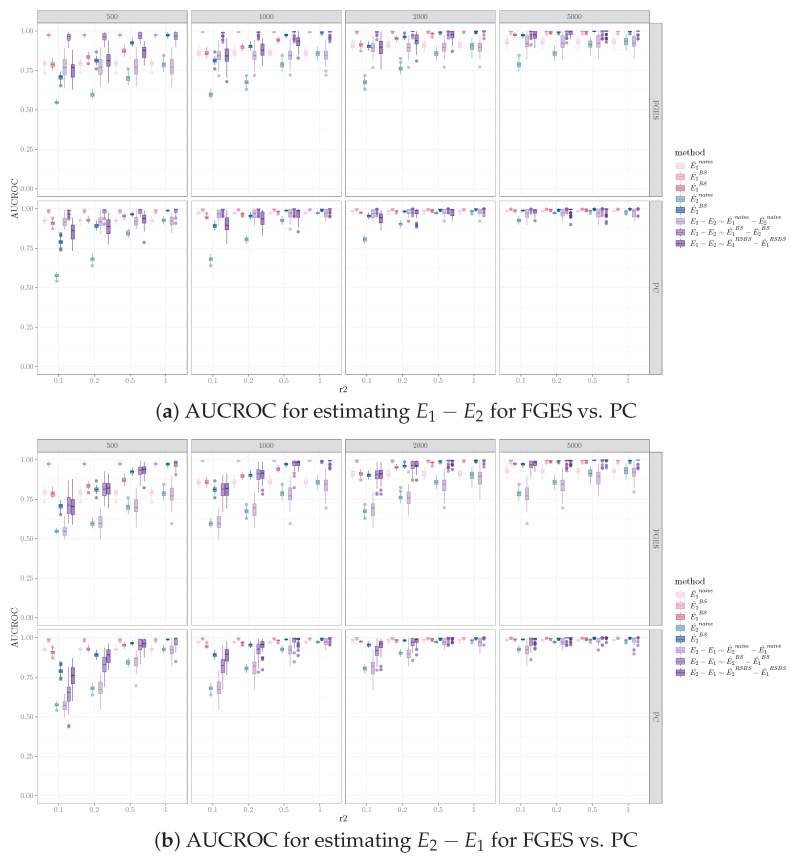
AUCROC for identifying orientation differences for $E_{1}-E_{2}$ vs. $E_{2}-E_{1}$ using FGES vs. PC, where $N_{v}=100$, $N_{e}=200$, $N_{d}=40$. Columns are sample sizes for $D_{1}$, and the x-axis represents the ratio of sample sizes for $D_{2}$ vs. $D_{1}$.

With respect to the interaction between the estimation methods and causal discovery algorithms, as indicated in Table 2, bootstrap is predominantly the best method for estimating network differences for both PC and FGES, except for when assessing the $E_{2}-E_{1}$ orientation performance. This indicates that there is an interaction between the causal discovery algorithm, the methods for estimating network difference, and the evaluated outcome. Figure 4 compares FGES and PC for a specific simulation setting to highlight the interaction effect.

### 5.5. Effect of Network Structure on Inferring Network Difference

In our analysis, we found the influence of network structure (i.e., the number of nodes and edges in $G_{1}$ and $G_{2}$ and the number of different edges between $G_{1}$ and $G_{2}$) to have minimal influence both on the numerical values of average AUCROC values and the relative advantage of the three methods for inferring network differences. Bootstrap estimation is predominantly the best method for inferring network difference for all variations of network structures. It demonstrated AUCROC values that were better than or statistically indistinguishable from the other estimation methods in >90% of the combinations of all pairs of sample sizes for $D_{1}$ and $D_{2}$, causal discovery algorithms, and evaluated outcomes. The mean AUCROC values across the different network structures were similar. As expected, the variability of the estimation decreased as the number of different edges increased.

**Figure 5:**
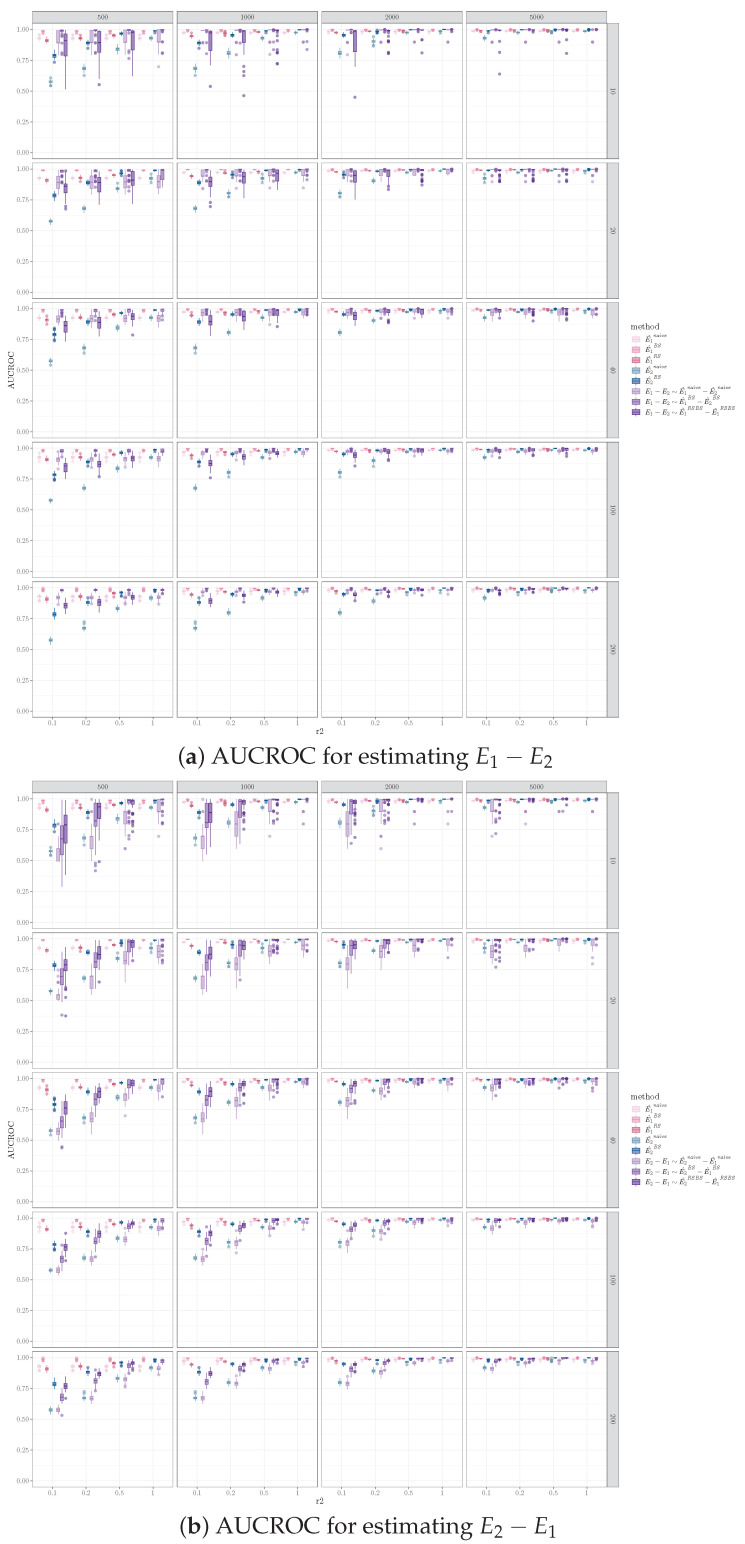
AUCROC for identifying orientation differences for $E_{1}-E_{2}$ vs. $E_{2}-E_{1}$ in different network structures using the PC algorithm, where $N_{v}=100$, $N_{e}=200$. Columns are sample sizes for $D_{1}$, the x-axis represents the ratio of sample size for $D_{2}$ vs. $D_{1}$. Rows are the number of different edges between $G_{1}$ and $G_{2}$.

For predicting $E_{1}-E_{2}$, the bootstrap method generally assigns a higher score than that of the equal sample size resampling for estimating $E_{1}-E_{2}$. On the other hand, for predicting $E_{2}-E_{1}$, the equal sample size resampling gives a higher score than that of the bootstrap method. This is because the two methods differ in how $E_{1}$ was estimated. The bootstrap method estimates $E_{1}$ with a larger sample size, compared to the equal sample size resampling method. This also explains the better AUCROC observed for $E_{2}-E_{1}$ with the equal sample size resampling method using the PC algorithm (Figure 5b).

### 5.6. Effect of Effect Size on Inferring Network Difference

**Figure 6:**
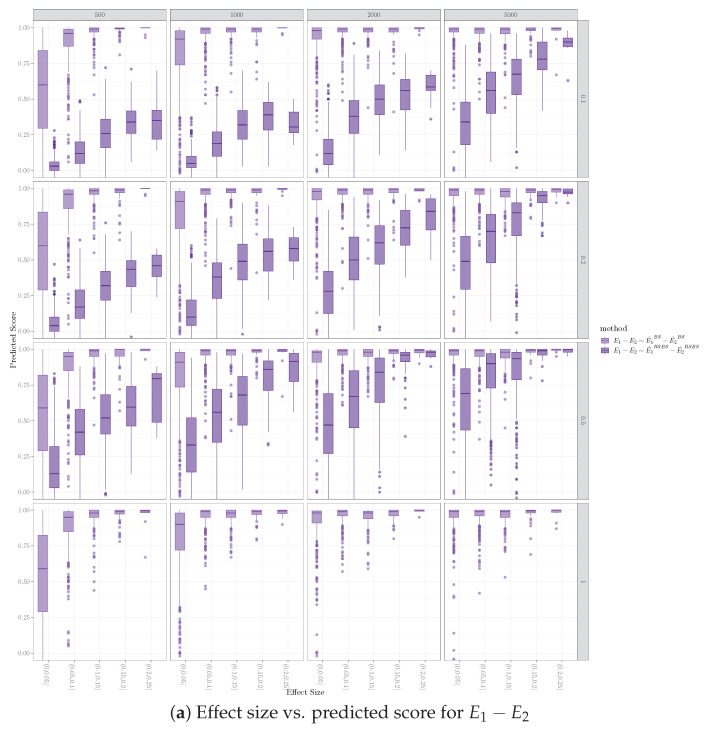

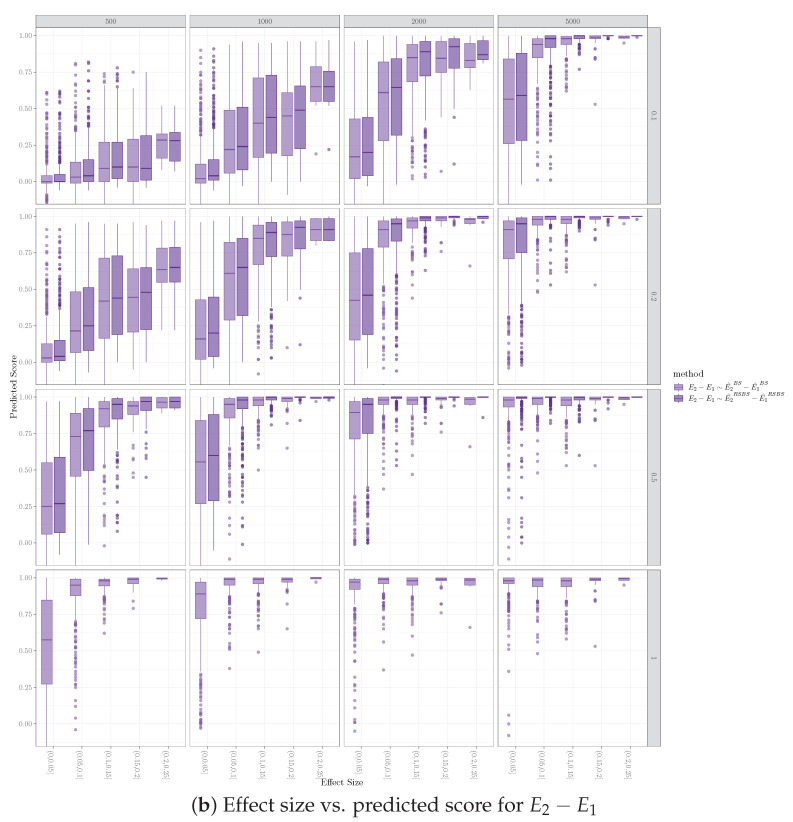
Effect of effect size on inferring network difference for orientation performance, where $N_{v}=100$, $N_{e}=200$, $N_{d}=40$, with PC algorithm. Each column represents a sample size for $D_{1}$, each row represents an r2 representing the sample size of $D_{2}$ over $D_{1}$. The x axis for each subplots represents the effect size, and the y axis represents the predicted score if there is an edge difference between the two graphs.

We examined the relationship between an edge’s effect size and its likelihood to be identified as different between the two graphs. Effect size refers to the strength of the relationship between pairs of variables (see Section 4 for definition). We used the predicted score for edge difference to represent the likelihood. The predicted score computed for the bootstrap and equal sample size resampling methods is specified in Equations (4) and (5). We observe that, given fixed sample sizes (e.g., individual subplots in Figure 6), edges with higher effect sizes receive higher predicted scores for edge difference, for both the bootstrap method and the equal sample size resampling method. As expected, when the sample sizes for $D_{1}$ and $D_{2}$ increased, the predicted scores for bootstrap and equal sample size resampling also increased.

## 6. Experiments with Real Data

To examine if the patterns observed from systematically simulated multivariate Gaussian data extend to that of the real world data, we selected six datasets from different domains of biology and medicine and applied the three methods for inferring network difference.

### 6.1. Experiment Design and Datasets

One challenge we faced is that the true causal Bayesian networks underlying the real world datasets are unknown. Therefore, we used the following strategy to generate $G_{1}$ and $G_{2}$: for each real world dataset $D_{0}$, we randomly selected two sets of variables of size $N_{p}$. We permuted the two sets of variables to generate the datasets $D_{1}^{f}$ and $D_{2}^{f}$, respectively. The superscript f indicates that $D_{1}^{f}$ and $D_{2}^{f}$ have the full sample size of the original dataset $D_{0}$. In theory, in the large sample, the operation of permuting a variable results in elimination of any edges connected to it. We then applied the causal discovery algorithm to $D_{1}^{f}$ and $D_{2}^{f}$, to obtain a pair of causal graphs, which we considered to be $G_{1}$ and $G_{2}$.

To evaluate the performances of the three methods for network difference inference, we applied the methods, given a subsample of $D_{1}^{f}$ and subsamples of $D_{2}^{f}$. We explored the following combinations for the experiments on real world data: (1) six real world datasets: as shown in Table 3, the real world datasets cover common experimental designs (clinical trials and cohort studies) and data modules (clinical data, biomarkers, electronic health record data, and high-throughput gene expression data) commonly seen in biomedical studies, containing a variety of sample sizes and numbers of variables. More information about these datasets and how to obtain them are included in the appendix. (2) $N_{p}$: $N_{p}=\left\{2,6\right\}$. For each $N_{p}$, 10 random repeats were conducted, resulting in 10 different pairs of $G_{1}$ and $G_{2}$. Note that the edge differences between each pair of graphs were generally not equal in number, as it depended on the connectivity of the variables that were permuted. (3) Sample sizes: for each $G_{1}$ and $G_{2}$ pair, we examined a subsample of $D_{1}^{f}$, which consisted of 60% of the observations from $D_{1}^{f}$. This sample size was referred to as $N_{1}$. For $D_{2}$, we examined the following sample sizes: $N_{2}=\left\{\left\lceil 0.1\times N_{1}\right\rceil ,\left\lceil 0.2\times N_{1}\right\rceil ,\left\lceil 0.5\times N_{1}\right\rceil ,1\times N_{1}\right\}$, similar to the simulated studies.

### 6.2. Causal Structure Discovery and Network Comparison

We used the FGES algorithm for all the real world datasets, since the PC algorithm did not terminate for the datasets with larger number of variables in a reasonable amount of time (up to 96 hrs per network for one combination of experimental parameters was allowed, due to the time constraint on the Minnesota Supercomputing Institute. It is worth noting, however, that parallelization can be implemented at the level of the resampling iterations for the bootstrap and equal sample-size resampling methods. We did not explore this in the current set of experiments). We examined the same performance measurements for the three methods for network difference inference as the simulated experiments.

**Figure 7:**
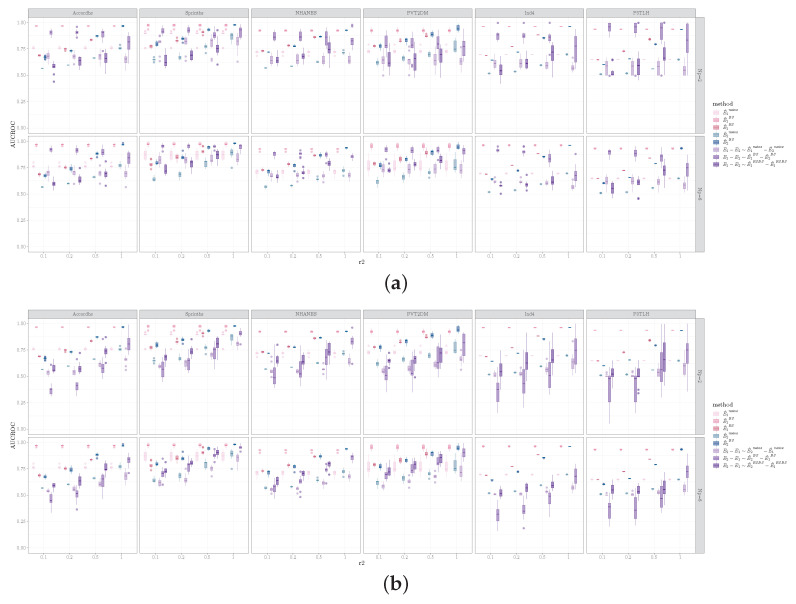
AUCROC for identifying (**a**) $E_{1}-E_{2}$ and (**b**) $E_{2}-E_{1}$ for the six real world datasets. Columns correspond to datasets, rows represent $N_{p}$, and the x-axis represents the ratio of sample sizes for $D_{2}$ vs. $D_{1}$.

**Figure 8:**
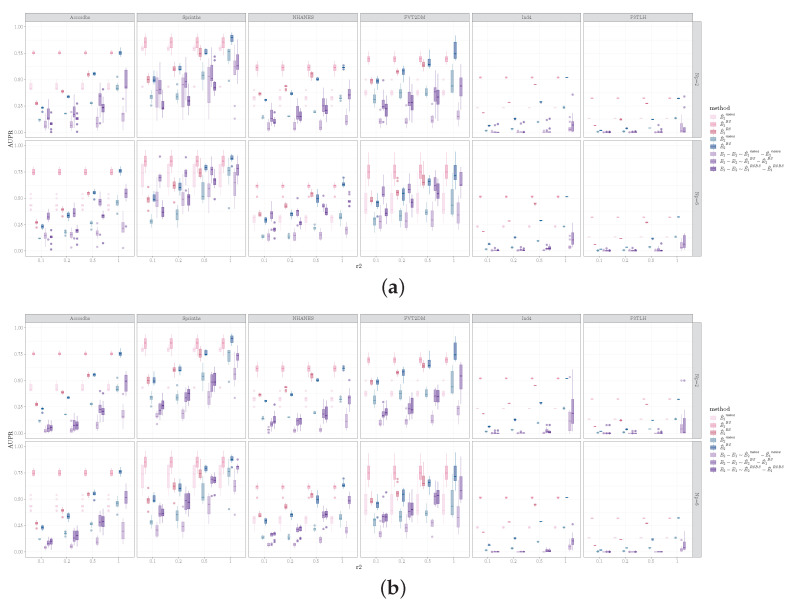
AUPR for identifying (**a**) $E_{1}-E_{2}$ and (**b**) $E_{2}-E_{1}$ for the six real world datasets. Columns correspond to datasets, rows represent $N_{p}$, the x-axis represents the ratio of sample sizes for $D_{2}$ vs. $D_{1}$.

Performances on the real world data are shown in Figure 7 and Figure 8. Notably, for the two single cell datasets (Ind4 and P3TLH), the performances were generally worse compared to the other datasets, except for the AUCROC inferring $E_{1}-E_{2}$ using the bootstrap method. This is likely due to the small sample to variable ratio for the single cell data.

The performances for the real-world data were somewhat different from what was observed for the simulated data. Comparing the estimation methods, the bootstrap method continued to perform the best when estimating $E_{1}-E_{2}$ with respect to AUCROC for all real world datasets. Contrarily, for estimating $E_{2}-E_{1}$, the equal sample size resampling method outperformed the bootstrap method for all datasets, in terms of AUCROC. For AUPR when estimating $E_{1}-E_{2}$, bootstrap was most frequently considered to be the best method, whereas, for AUPR when estimating $E_{2}-E_{1}$, the equal sample size resampling method had similar performance to the bootstrap method and outperformed bootstrap for several dataset and sample size combinations.

It is interesting to note that, when estimating $E_{1}-E_{2}$, we observed that, for most datasets, as the sample size for $D_{2}$ increased, the AUCROC for the bootstrap estimation for edge difference decreased, despite the increase in performance for estimating $E_{2}$. Upon further examination of the results, we discovered that this was due to the bootstrap estimation for $E_{2}$ tending to assign a higher score to edges as the sample size of $D_{2}$ increased. This, in turn, resulted in the assignment of lower scores for positives when evaluating $E_{1}-E_{2}$. We did not observe this in the simulated datasets. It is likely due to the difference in the distributions of the real world data vs. the simulated data.

## 7. Key Findings and Recommendations

- The sample size of the smaller datasets impacted the performance more compared to the total sample sizes from both datasets. When planning data collection with the goal of identifying different causal relationships between two populations, aim for maximizing the minimal sample size.
- The naive method is not recommended for inferring network differences due to its suboptimal performance for AUCROC, AUPR, and cross entropy in most of our simulated and real-world data experiments.
- With the default parameterizations, the PC algorithm outperforms the FGES algorithms in most simulated experimental conditions. The PC algorithm is therefore recommended over the FGES for inferring network differences for data distributions similar to our simulation experiments.
- In both our simulated and real-world data experiments, we observed that the relative effectiveness of the bootstrap vs. the equal sample size resampling methods depended on other factors (e.g., the causal discovery algorithm applied and if $E_{1}-E_{2}$ or $E_{2}-E_{1}$ was estimated).
- The real-world data experiments displayed different behaviors compared to the simulated data experiments, potentially due to their more complex data distributions. The choice of method for estimating network differences for a specific pair of datasets should be informed by simulation experiments that approximate the datasets in question.

## 8. Discussion and Future Work

The contributions of the current work are as follows: (1) we provided the mathematical formulation for the problem of estimating causal Bayesian network difference. (2) We introduced three methods for inferring the structural difference between pairs of causal Bayesian networks. (3) Finally, we evaluated the performances of the three methods with systematically designed simulations and a wide range of real-world biomedical data.

Given the results, we recommend against using the naive method for inferring network structural differences, especially when the two datasets in question differ substantially in sample size. This recommendation is both due to the inferior performance of the naive method and its inability to capture the uncertainty or confidence of the inference. In both the simulated and real-world data experiments, we observed that the bootstrap method outperformed the equal sample size resampling method for inferring $E_{1}-E_{2}$ when $D_{1}$ has larger sample sizes. In the simulated experiments, the bootstrap method outperformed the equal sample size resampling method for inferring $E_{2}-E_{1}$ in some conditions. However, in the real-world data experiments, the equal sample size resampling method outperformed the bootstrap method for inferring $E_{2}-E_{1}$ in all conditions. The real-world data experiments displayed different behaviors compared to the simulated data experiments, potentially due to their more complex data distributions. The choice of method for estimating network differences for a specific pair of datasets should be informed by simulation experiments that approximate the datasets in question.

The simulation portion of this work provided a flexible and expandable framework for evaluating methods for inferring causal Bayesian network differences. We focused our attention on multivariate Gaussian distributions generated by sets of linear equations (structural equation models) constrained by the causal structure, but other data distributions and data generation protocols can be readily incorporated. Similarly, any causal discovery methods can be used as the base method for causal structure discovery in place of FGES and PC. Further, we focused on performance measurements that characterized the quality of global structural discovery. Additional performance measurements can be added to evaluate other aspects of network differences. For example, if one is interested in the structural difference around a specific variable or a specific set of variables, instead of using the metrics computed over the entire causal Bayesian network as in the current study, the metrics can be computed on the subgraph of interest. Another task that might be of interest to practitioners is to estimate the differences in the causal effect between a pair of variables in different causal Bayesian networks. This is a more involved task, since the estimated causal effect depends on the estimated causal structure, and error can occur in both estimation steps. On the high level, estimating causal effect difference can be achieved by adding an additional step of effect estimation following the causal structure discovery to generate the estimated causal effect differences, and using metrics to evaluate the similarity of continuous quantities (with one example being the structural intervention distance proposed in Ref. [29]) to assess the alignment of the true vs. estimated causal effect difference.

It is also worth noting that, although our simulated data experiments were designed to evaluate the performance of the three methods for network difference inference, they can be easily repurposed for sample size estimation when the researchers are planning data collection with the goal of contrasting the causal mechanisms under distinct conditions. To estimate the proper (e.g., minimally acceptable) sample sizes for the two datasets, the researchers can parameterize the two causal Bayesian networks given prior domain knowledge (e.g., edge density, strength of edges, and the expected structural difference between the networks), generate datasets with different sample sizes, and apply the network difference inference methods of their choice to evaluate the performance. The sample sizes can be determined by picking a threshold on one or more performance metrics (e.g., the sample sizes that resulted in AUCROC ≥0.8).

In conclusion, this study serves as an important first step for the development of more comprehensive causal Bayesian network difference inference methods.

## Figures and Tables

**Table 1:** Causal structure discovery results using the three different methods on the network pair $\left(G_{1},G_{2}\right)$ in Figure 1. Sample size for $D_{1}$ is 1000, sample size for $D_{2}$ is 200. Edges with zero value for $\hat{E_{1}}$ or $\hat{E_{2}}$ for all methods are omitted. GS indicates the gold standard, i.e. if the edge is different between $G_{1}$ and $G_{2}$ in terms of the causal skeleton.

	GS	Naïve			BS			RSBS		
Edge	$E_{1}-E_{2}$	$\hat{E_{1}}$	$\hat{E_{2}}$	$\hat{E_{1}}-\hat{E_{2}}$	$\hat{E_{1}}$	$\hat{E_{2}}$	$\hat{E_{1}}-\hat{E_{2}}$	$\hat{E_{1}}$	$\hat{E_{2}}$	$\hat{E_{1}}-\hat{E_{2}}$
D—E	1	1	0	1	0.94	0	0.94	0.42	0	0.42
A—C	0	1	0	1	0.86	0.1	0.76	0.16	0.1	0.06
B—C	0	1	1	0	1	1	0	1	1	0
C—D	0	1	1	0	1	1	0	1	1	0
A—D	0	0	0	0	0.04	0.02	0.02	0.04	0.02	0.02
B—E	0	0	0	0	0.24	0	0.24	0.04	0	0.04

**Table 2:** Summary of relative performance for estimating network difference using the three estimation methods. The table summarizes the percentage of times a network difference estimation methods resulted in the best performance compared to the other two; if a methods’ performance was not statistically distinguishable from that of the best one (defined as being within one standard deviation), it was also marked as the best. The determination of the best method was conducted for each simulation condition, evaluated outcome, and performance measure. For the naive method and the bootstrap (BS) method, the percentages reported in the table were computed based on 240 simulation conditions ($|N_{v}\left|\;\times \;\right|N_{e}\left|\;\times \;\right|N_{d}\left|\;\times \;\right|N_{1}\left|\;\times \;\right|N_{2}|\;=240$). For the equal sample size resampling (RSBS) method, the percentages reported in the table were computed based on 180 simulation conditions, since this method was not applicable when $N_{1}=N_{2}$. With respect to the evaluated outcome, we assessed both the performance of skeleton discovery and orientation discovery. $E_{i}-E_{j}$ refers to edges in $G_{i}$ but not in $G_{j}$, based on $D_{i}$ and $D_{j}$. In our experiments, the sample size of $D_{1}$ was always larger or equal to that of $D_{2}$. We present the results for $E_{1}-E_{2}$ and $E_{2}-E_{1}$ separately. It is worth noting that, the bootstrap method resulted in the best performance over almost all simulation conditions for almost all evaluated outcomes, algorithms, and performance measures. The only exception is when assessing the additional oriented edge in the network estimated from a smaller dataset as compared to the network estimated from a larger dataset using the PC algorithm for the AUCROC (underlined).

Estimation Method	Evaluated Outcome		Discovery Algorithm	AUCROC	AUPR	Cross Entropy
Naïve	$E_{1}-E_{2}$	Skeleton	FGES	5%	3%	0%
			PC	8%	1%	0%
		Orientation	FGES	0%	8%	0%
			PC	5%	1%	0%
	$E_{2}-E_{1}$	Skeleton	FGES	5%	10%	0%
			PC	7%	5%	0%
		Orientation	FGES	0%	10%	0%
			PC	3%	5%	0%
BS	$E_{1}-E_{2}$	Skeleton	FGES	100%	98%	97%
			PC	100%	94%	93%
		Orientation	FGES	100%	100%	98%
			PC	100%	100%	100%
	$E_{2}-E_{1}$	Skeleton	FGES	100%	100%	100%
			PC	99%	100%	99%
		Orientation	FGES	100%	100%	100%
			PC	74%	100%	99%
RSBS	$E_{1}-E_{2}$	Skeleton	FGES	8%	59%	56%
			PC	52%	82%	79%
		Orientation	FGES	21%	63%	78%
			PC	3%	1%	0%
	$E_{2}-E_{1}$	Skeleton	FGES	100%	58%	39%
			PC	100%	100%	100%
		Orientation	FGES	100%	100%	100%
			PC	100%	78%	74%

**Table 3:** Descriptions of the real world datasets.

Name	# Obs	# Var	Description	Citation
Accordbs	10,251	70	Baseline data from the ACCORD clinical trial	[49]
Sprintbs	9361	27	Baseline data from the SPRINT clinical trial	[50]
NHANES	20,044	65	Lab data from the NHANES3 cohort study	[51]
FVT2DM	79,486	33	EHR data from a type 2 diabetes cohort from Fairview hospital	[52]
P3TLH	2621	1948	Single cell gene expression data from Hepatocytes from patient P3TLH	[53]
Ind4	3982	1462	Single cell gene expression data from breast epithelial cells from individual 4	[54]

## Data Availability

Codes for generating the simulated data are provided. To obtain the real-world dataset, please contact the original authors that generated the datasets.

## Supplementary Materials

The following supporting information can be downloaded at: https://www.mdpi.com/article/10.3390/e26030228/s1. Table S1: distribution of causal effect sizes for simulated data; Table S2: comprehensive results of the simulated data experiments; Table S3: comprehensive results of the real-world data experiments; Table S4: comparisons of different estimation methods and causal discovery algorithms in different simulation conditions; R codes for generating the simulated data and analysing the results.
